# Residents as teachers in Neurology: a Germany-wide survey on the involvement of neurological residents in clinical teaching

**DOI:** 10.1186/s42466-022-00170-3

**Published:** 2022-05-09

**Authors:** Anne-Sophie Biesalski, Lars Tönges, Isabelle von Kirchbauer, Eileen Gülke, Hanna Eisenberg, Franziska Maria Ippen, Friederike Schmidt-Graf

**Affiliations:** 1 Department of Neurology, Ruhr-University Bochum, St. Josef Hospital, Gudrunstraße 56, 44791 Bochum, Germany; 2 Center for Protein Diagnostics (ProDi), Ruhr University Bochum, Bochum, Germany; 3 TUM Medical Education Center, Technical University of Munich, Munich, Germany; 4 Department of Neurology, University Medical Center Hamburg-Eppendorf, Hamburg, Germany; 5 Department of Neurology, Universität Medical Center Göttingen, Göttingen, Germany; 6 Department of Neurology, University Hospital Heidelberg, Heidelberg, Germany; 7 Department of Neurology, Technical University of Munich, Munich, Germany

**Keywords:** Residents-as-teachers, Residency, Neurology, Teaching, Medical education

## Abstract

**Background:**

Residents play an important role in the clinical training of medical students, spending up to 25% of their daily work teaching. In the US medical curriculum didactic courses for residents already exist and their role as a teacher is firmly anchored. In Germany, there are no fixed regulations or residents-as-teachers-programs. The aim of this study was to evaluate the activities of neurological residents in clinical teaching.

**Methods:**

We conducted a prospective cross-sectional online survey among neurological residents in Germany. The evaluation was carried out descriptively and by means of text analysis.

**Results:**

138 residents from 39 German neurological university hospitals answered the survey. Nearly half of them needed the teaching activity as part of their career planning. The residents are mostly involved in practical courses. More than 80% stated, that they enjoy teaching. 64% stated that there were no preparatory courses for teaching at their hospital/university. 78.4% of the respondents received no or merely insufficient feedback for their own teaching and 62.5% had only little or even no knowledge about the university curriculum.

**Conclusions:**

By teaching medical students, residents play an outstanding role in recruiting students for neurology and, simultaneously, teaching leads an improvement in the residents’ own learning. To encourage young neurologists as teachers and—at the same time as learners—Clinic directors and universities should promote residents-as-teachers programs in neurology and reward the residents’ teaching activities.

**Supplementary Information:**

The online version contains supplementary material available at 10.1186/s42466-022-00170-3.

## Introduction

German medical education constantly develops towards more practical innovative training programs [1]. As part of these model study programs, residents play a central role in students’ medical education. In their daily work, they are extensively involved in clinical teaching, especially in bedside-training courses as well as one-to-one education in the clinical context. Medical residents spend up to 25% of their daily work teaching medical students, other residents and non-medical staff [2]. Furthermore, medical students stated that they obtained approximately a third of their knowledge from residents, which is more than the influence of any other faculty member [3].

The great importance of this educational aspect has been recognized widely and teaching by residents—especially in the USA—has been incorporated into many educational programs and/or has been set as a standard [4]. The American Academy of Neurology (AAN) has firmly anchored the role of the resident as a teacher in its core curriculum guidelines [5].

Especially in North America and Canada, numerous teaching courses for residents have been created [3, 6].

While the skill “*teaching”* is already embedded in the fourth study year in the USA, there are very few guidelines about this subject in Germany. The training of teaching-methods is not incorporated in the currently valid licensing regulations or in the drafted bill of the new version. The new version even states that residents may only be included in teaching after they have completed the third year of further training [7]. However, in the national catalogue of learning objectives (Nationaler Kompetenzbasierter Lernzielkatalog Medizin, NKLM), teaching is included for the fourth and fifth study year [8]. Interestingly, the subject of teaching is not mentioned in the guidelines of the German Medical Association, listing the national content of postgraduate medical education.

Despite the discrepancies stated above, it is unquestionable that residents in all departments are involved in the daily clinical teaching of students. The objective of this study was to evaluate how residents in neurology are prepared for teaching and whether guidelines exist in Germany on how to teach.

## Methods

This is a prospective cross-sectional study of residents in neurology to analyze the implementation of teaching and their daily involvement in it.

The German neurological Society (DGN) founded a commission about neurological teaching in 2020. In cooperation with the resident fellow section of the DGN (*Junge Neurologen*), a task force was initiated to investigate how neurological residents are prepared for teaching medical students. The group developed an online questionnaire that was sent to residents of all neurological university hospitals in Germany in May and June 2021. The questionnaire contained 15 questions about the implementation of neurological teaching and how residents were involved in it. In addition, the residents' satisfaction and interest in teaching were measured using a Likert scale (see Additional file 1).

After a five-week survey period, 138 residents from 39 university hospitals who had answered the questionnaire were included for further analysis. The subsequent evaluation was done using EvaSys®. Standard descriptive statistics were used to analyze the Items A-C, the text fields (D/E) were evaluated qualitatively and categorially using MS-Excel®.

## Results

138 residents (79 female, 58 male) from 39 German neurological university hospitals answered the survey. Mean age was 32 years. Most of them were in their 3rd to 4th or later years of further training (Table 1: Baseline characteristics).

**Table 1:** Baseline characteristics

Item					n (total)
A 1: Gender	Male 42.3% (n = 58)	Female 57.7% (n = 79)	Diverse 0% (n = 0)		137
A2: Age	up to 30 years 44.5% (n = 61)	31–35 years 43.1% (n = 59)	36–40 years 10.2% (n = 14)	> 40 years 2.2% (n = 3)	137
A3: Year of specialist training	1–2 28.3% (n = 39)	3–4 36.2% (n = 50)	> 5 35.5% (n = 49)		138

43.8% stated that they needed the teaching activity as part of their career planning, like for their habilitation or to qualify for academic tenure. 57.2% reported that they had studied at the same university and were therefore familiar with the structure and content of the given course. 83.2% were scheduled for courses nearly or every semester. The average teaching time of the residents was around 7.1 h per semester (this corresponds to one semester weekly hour) and the residents are mostly involved in practical courses such as bedside teaching, internships, courses during the practical year in Germany in the last year of medical school and compulsory seminars. The preparation time was given as 1–2 h per semester. 62.7% of the participants stated that they felt sufficiently prepared for clinical teaching.

Support from superiors: Regarding the question of didactic support in the planning and implementation of courses, 64% of the residents stated that there were no preparatory courses for teaching at their hospital/university. Of the 36% with existing preparatory courses at their institution, 24 gave free text information on specific offers in teaching. Of these, 19 residents reported lecturer training courses for teachers. Moreover, two specific problem-oriented learning (POL) introductory seminars were named, teaching materials such as teaching videos or online courses were listed three times and also three residents stated that their faculty supports a participation in the Master of Medical Education program (MME).

Feedback on teaching skills: 78.4% of the respondents received no or merely insufficient feedback for their own teaching abilities. 40% of them did not know whether there was a specific award for good teaching at their faculty and 62.5% stated that they had only little or even no knowledge about the university curriculum. Only 35.3% of the residents were informed about the content and timetable of exams in neurology at their own faculty. 77.2% stated that they were interested in a teaching webcast or online training on teaching.

80.9% of the respondents answered the question “*I enjoy teaching”* with “I agree” or “I fully agree”. The statement “*teaching is a burden for me”* was mostly answered neutrally by the participants (Table 2).

**Table 2:**
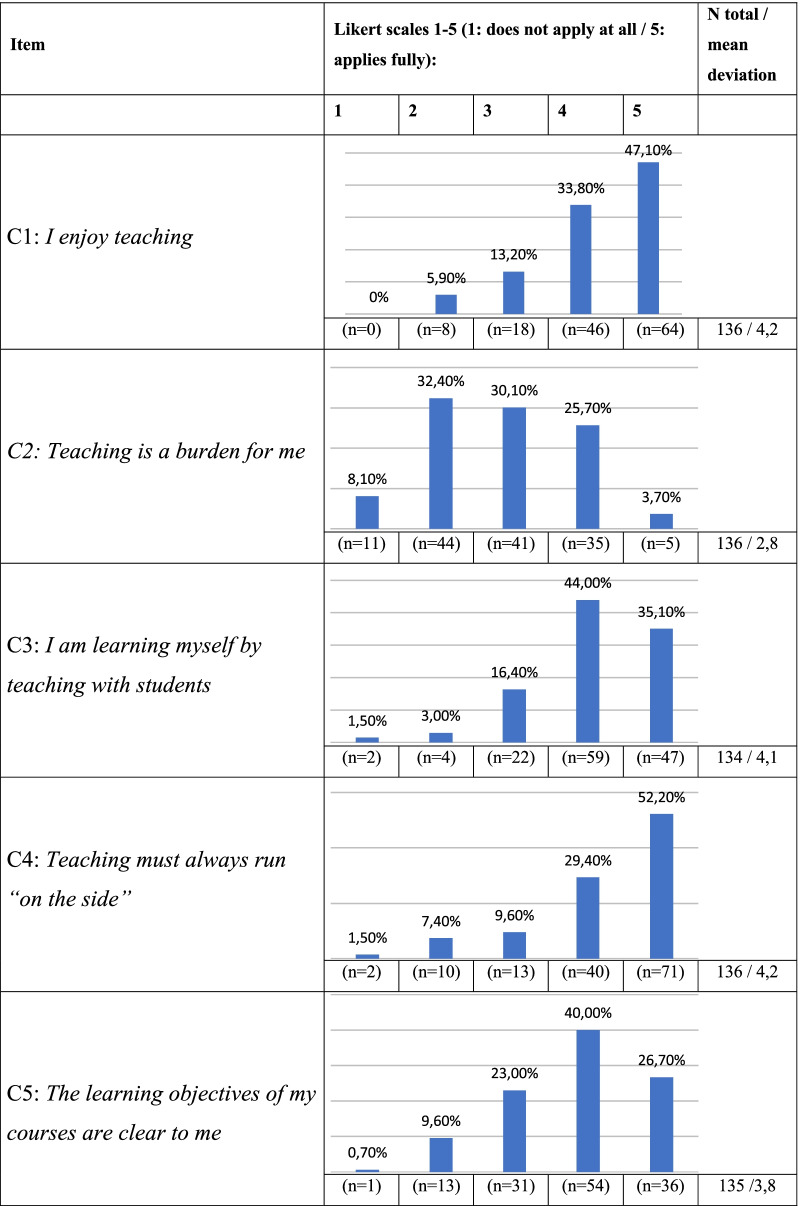

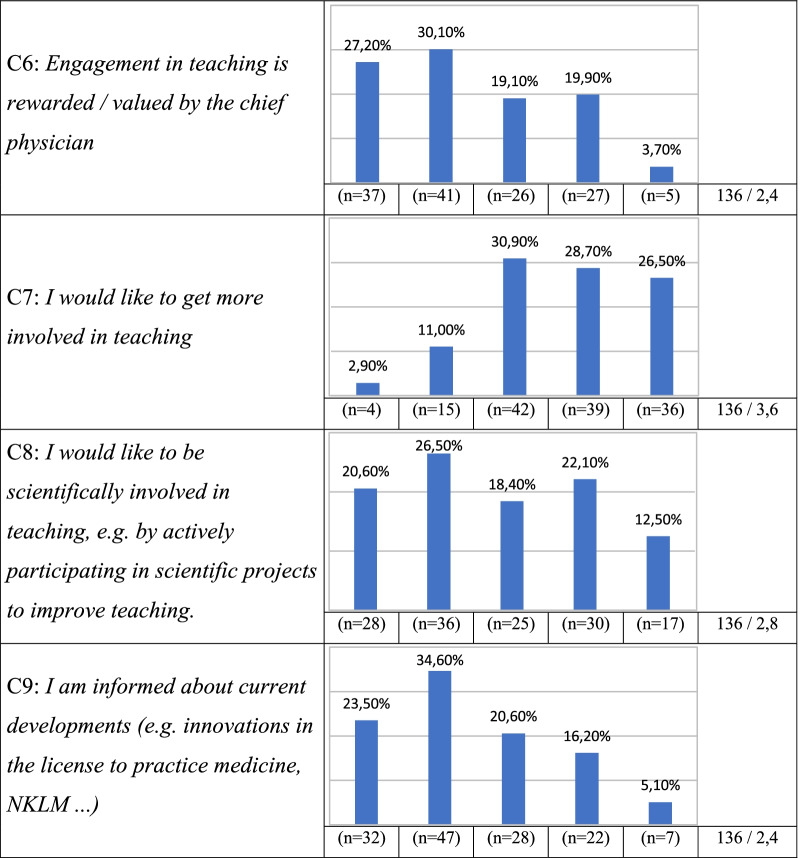
Statements from the Likert scale

Teaching in times of COVID-19: Regarding the question of changes in the context of the COVID-19 pandemic, 82 items of information were provided in free text fields. It was predominantly stated that the teaching within the framework of the corona restrictions was carried out entirely or at least predominantly online. Six participants mentioned the increased workload resulting from the online changeover as well as teaching outside of working hours. Four participants stated that the quality of practical on-site lessons had increased due to the reduction of group sizes.

Seven respondents left an additional statement at the end of the survey. 2 of the statements related to the interest in being involved in clinical teaching (e.g. *“I am very interested in being a tutor”*). The other 5 free texts related to a lack of appreciation and attention to teaching in everyday clinical practice (e.g. *“Teaching must finally be recognized as an additional activity for which sufficient (!!) time must be planned including preparation and follow-up …”*).

## Discussion

This study provides an insight into how neurological teaching in Germany is carried out from a residents’ perspective. Regarding the relatively short time period of five weeks and the exclusive addressing of university hospitals, a proportionately reasonable number of residents responded to the survey. (A brief note on the representativeness may be found in Additional file 2).

Although the authors believe that this is an important and thus far neglected topic, there is unfortunately little literature about teaching by neurological residents, which made it difficult to compare the data obtained in this study.

### Teaching and workload

Most of the residents enjoy clinical teaching and are motivated in getting more involved in this field. The additional workload arising from the planning and implementation of a student course is relatively low, but needs to be done in addition to regular work. Due to the already existing workload of neurological residents with a high proportion of administrative activities and frequent overtime work [9], it has to be considered that the interest in teaching might decrease. Residents spend up to a third of their working time with teaching students [10, 11]. In addition, residents play a key role in the training of medical students, which has been shown in previous studies [12]. However, apparently no concepts for a systematic teaching by residents exist in Germany. Overall, residents are apparently not given sufficient recognition for their work in teaching and do not receive enough feedback in this area [9], a fact which can be also shown in our data. Besides, they do not seem to be sufficiently informed about the learning objectives of their students: More than 60% of the participants do not properly know the curriculum and almost a third of them is not informed about the type or the expected horizon of exams in neurology. Regarding the aim of constructive alignment in neurological teaching, there is an urgent need for proper communication of the learning objectives in the departments themselves to ensure that goals of the curriculum are met and neurological teaching is done homogenously.

One possible reason for the lack of interest by superiours seems to be the allocation of funds. Funding is rather difficult to receive for teaching and the performance-based allocation of funds continues to predominantly reward classical clinical or laboratory-based research. The performance-oriented allocation of funds in teaching (LOM: *Leistungsorientierte Mittelvergabe*) is mainly regulated by the faculties themselves, and incentives such as awards for good clinical teaching do not exist at all university hospitals [13]. As a result, this may also reduce the attractiveness of clinical teaching.

LOM points are weighted for each lecture, seminar, bedside course etc. depending on the time required, the number of students and the type of lesson. However, this does not always reflect the actual effort and importance of the teaching. For each LOM point, the respective department receives a financial equivalent. Nevertheless, the allocation of funds is still very heterogeneous among the various faculties [14, 15].

Residents participating in our survey had mostly not been informed about current developments in medical teaching in Germany (i.e. Referentenentwurf zur neuen Approbationsordnung, NKLM). Chair holders in particular should inform their employees about political developments in teaching and curricular changes and should encourage them to exert influence on it.

### Educational research

The importance of good teaching in order to recruit motivated employees still does not seem to be recognized at some places, which is reflected in the analyzed items B4-7 and C4-7 in our study. This is also shown by the fact that findings from educational research find their way into clinical teaching slowly, although the research and implementation of modern teaching methods is of enormous importance for our subject [16]. With reference to the redesigning of medical curricular enforced by the COVID-19 pandemic, university hospitals in which a good teaching infrastructure and innovative, digital teaching methods already existed, had less difficulty adapting to the changes [17].

### To teach is to learn!

Teaching improves one’s own learning. Also medical teachers may gain more knowledge by teaching than their own learners [18, 19]. Accordingly, the residents in our study stated to learn better by teaching. The question arises why *teaching* is not consciously used as a part of residents further training, since formats such as peer teaching and tutorials have been recognized as a teaching method at universities for decades. Despite the fact that 80% of final-year medical students state that they are interested in teaching [20] there are hardly any opportunities to receive didactic training in Germany. The fact that young residents benefit from teaching is even further neglected by the new version of the German licensing regulations, stating that young residents should not be given the opportunity to teach during the first three years of their postgraduate training at all. These circumstances make it significantly harder for residents to improve their teaching skills from the very beginning and they do not get access to improve their own knowledge by contributing to the curriculum. However, the fact that even residents at their very early stages of career are involved in teaching is—regardless of the new version of the German licensing regulations—a fact that incidentally cannot be reconciled with clinical reality, as shown in our study.

### Positive effects of resident-as-teacher programs

64% of the neurology residents in our study have not yet participated in a teaching course at their university. However, the positive effects of resident-as-teacher programs have been proven in several studies by now [4], even if there is a lack of teaching-programs in neurology. In addition to the residents’ benefit from their own teaching [18], structured teaching programs increase job satisfaction [21] and lead to improved patient care [22].

Last but not least, teachers have a role model function for students and are often important advisors for their professional careers. Students who had experienced a good neurological teaching will possibly choose this subject as their later profession. Teaching-courses may also influence the decision of residents for future career choices [4]. Therefore, excellent clinical teaching in neurology has a significant influence on the recruiting of future neurologists. And this is of main importance in times of demographic change and the growing therapeutical opportunities in our field.

In summary, the positive effect of residents as teachers overweighs potential disadvantages and can result in satisfied, enthusiastic residents contributing not only to young talent acquisition in this field, but also to improved patient care. However, this subject is still relatively neglected.

The myth that teaching (or teaching to teach) takes too much time has been refuted: Usatine et al. analyzed the time that an experienced teacher spends on teaching students at the bedside with the time that he or she needed alone with the patient. There were no significant differences as to whether teaching took place or not [23]. In order to achieve this result, however, it is important to train teachers and especially residents in bedside teaching.

### How should teaching be taught?

The question is how teaching by residents in neurology can be promoted and improved. In addition to improving the communication of learning objectives and exam content and political developments (e.g. new version of the licensing regulations for the medical education), medical didactic centers in particular should increasingly approach residents and offer didactics courses.

There are a number of resident-as-teacher activities that have also been integrated into an educational program for neurology-residents based on the *6 principles of adult learning theory (ALT)* [24]. However, as far as we know, there are very few purely neurological resident-as-teacher programs [12]—and we do currently not have any information on such an existing program in Germany.

**Fig. 1:**
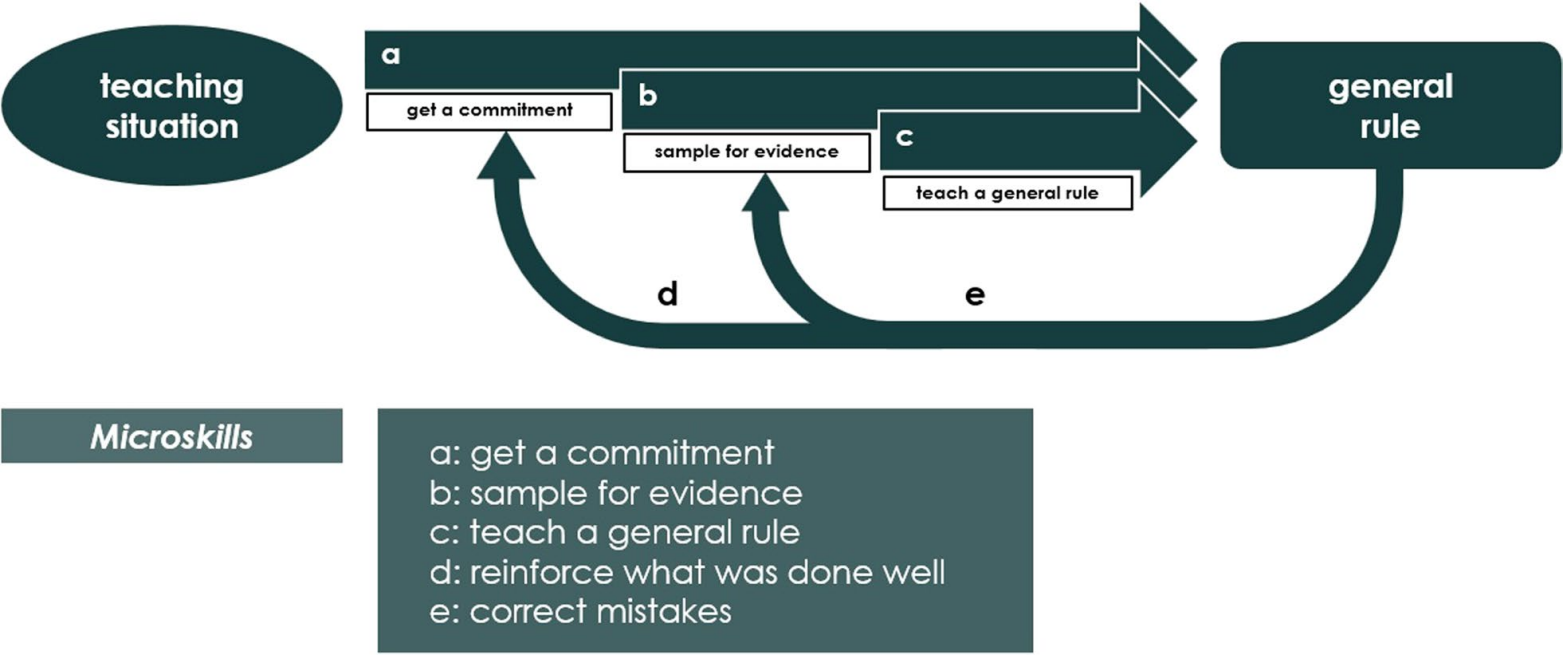
Technique of the five-step Microskills Model [25, 26]

Nonetheless, some teaching methods that have been described are easy to train and can therefore also be used in German neurological clinics. These methods include the technique of the five-step Microskills Model [25, 26], or “One-Minute Preceptor”, which was developed in 1992. It has the goal to improve teaching efficacy and efficiency. The five-step structure contains: (a) get a commitment; (b) sample for evidence; (c) teach a general rule; (d) reinforce what was done well; and I correct mistakes [27] (Fig. 1).

Frank and Józefowicz also gave a good overview of different teaching methods in neurology (e.g. grand rounds, patient-oriented bedside-teaching, Case-based small-group teaching, work rounds etc.) and described how important enthusiasm and commitment are in neurological teaching [22].

It is also worthwhile to be informed about current teaching methods, i.e. by using platforms such as MedEdPORTAL [28] and the German Society for Medical Education (GMA) [29]. It would also be desirable to have separate information websites for the respective medical faculties, for example through the department for medical didactics, and the existence of such a department is therefore of considerable value for each medical faculty.

### Limitations of this study

One limitation of this study is that, in order to preserve anonymity, we did not explicitly ask for the location of the hospital/university and therefore cannot determine whether all university hospitals participated.

## Conclusion

Motivated teaching ensures motivated employees. This fact should be recognized particularly by medical directors and chair holders. The engagement of residents in teaching is high and there is a great interest in further development in this area. Chief physicians can benefit from this requirement in several ways: By releasing employees to prepare for teaching, the quality of the courses will increase, and residents will benefit from teaching in their own ongoing/continuing training. Overall, engaged teaching will attract motivated future employees, who will increase the quality of clinical work.

This study led to the summary of the following conclusions. Politics should lay the foundation to anchor teaching—also by young residents—in the new version of the license to practice medicine in order to establish teaching as a mandatory cornerstone of academic education. Secondly, managers of the university hospitals have an increasing responsibility to recognize the enormous potential of “residents-as-teachers”—and to take advantage to improve the residents’ education and patient care.

## Supplementary Information

**Additional file 1.** Attachment 1: Questionnaire.

**Additional file 2.** Supplementary information.

## Data Availability

The datasets generated during and/or analyzed during the current study are available from the corresponding author on reasonable request.
