# Supplementary material for: Residents as teachers in Neurology: a Germany-wide survey on the involvement of neurological residents in clinical teaching
Source: Neurol Res Pract. 2022 May 9;4:17. doi: 10.1186/s42466-022-00170-3 (PMC9080961; doi:10.1186/s42466-022-00170-3)
Supplement: Supplementary file 1 — Additional file 1. Attachment 1: Questionnaire. [file 42466_2022_170_MOESM1_ESM.docx]

**Attachment 1: Questionnaire**

(A)

A1: Gender: m / f / d

A2: Age: up to 30 years / 31-35 years / 36-40 years /> 40 years

A3: Year of specialist training: 1-2 / 3-4 / 5 or more years

A4: I need the teaching activity for my habilitation or similar: yes / no

A5: I also studied at my university hospital location and therefore know the processes and

timetables: yes / no

(B)

B1: I am assigned to teaching almost every semester: yes / no, seldom / no, never;

If so, how many hours (60 minutes each) of pure teaching time per semester: 1-3 hours / 4-8

hours / 8-12 hours / 13 hours and more

B2: In which teaching formats are you involved: Lecture / practical course such as bedside,

block internship etc / compulsory seminar / voluntary seminar event / practical year lessons

/ doctoral seminar / other: (free text)

B3: My preparation time for teaching is: <1 h / 1-2 h / 3-5 h /> 5 h per semester

B4: I feel sufficiently prepared for teaching: yes / no;

B5: Do you have the opportunity to take part in courses that support / prepare you for teaching

(e.g. didactic courses)? No / Yes; if so, what? (Free text)

B6: Do you receive feedback / evaluations for your teaching? Yes, sufficient / yes, insufficient /

no

B7: Is there a prize/ incentive for good or innovative teaching at your location? Yes, one / Yes,

several / No / I don't know

B8: Do you know the study procedures / curriculum at your university? Yes / just about / no

B9: Do you know how the neurology students are examed at your location? Yes / roughly / no

(C)

Likert scales 1-5 (1: does not apply at all / 5: applies fully):

C1: I enjoy teaching

C2: Teaching is a burden for me

C3: I am learning myself by teaching with students

C4: Teaching must always run "on the side"

C5: The learning objectives of my courses are clear to me

C6: Engagement in teaching is rewarded / valued by the chief physician

C7: I would like to get more involved in teaching

C8: I would like to be scientifically involved in teaching, e.g. by actively participating in

scientific projects to improve teaching.

C9: I am informed about current developments (e.g. innovations in the license to practice

medicine, NKLM ...)

(D)

Others:

D1: Are you interested in a teaching webcast / online training or similar? Yes / No;

D2: other wishes / suggestions: (free text)

(E)

Corona special

E1: What has the corona pandemic changed for you in teaching at your location? (Free text)
