# Supplementary material for: Residents as teachers in Neurology: a Germany-wide survey on the involvement of neurological residents in clinical teaching
Source: Neurol Res Pract. 2022 May 9;4:17. doi: 10.1186/s42466-022-00170-3 (PMC9080961; doi:10.1186/s42466-022-00170-3)
Supplement: Supplementary file 2 — Additional file 2. Supplementary information. [file 42466_2022_170_MOESM2_ESM.docx]

**Residents as teachers in Neurology: A Germany-wide survey on the involvement of neurological residents in clinical teaching**

Anne-Sophie Biesalski^1^, Lars Tönges^1,2^, Isabelle von Kirchbauer^3^, Eileen Gülke^4^, Hanna Eisenberg^5^, Franziska Maria Ippen^6^, Friederike Schmidt-Graf^7^

**Supplementary Information**

Since we do not know the exact number of residents who are involved in teaching in Germany, we had to estimate the total number: Our internal research has shown that on average 38 residents work in the neurological university hospitals in Germany. Estimated that half of them are constantly active in inpatient care and thus mainly in bedside teaching, this would account for 19 residents per hospital. With a total of 39 inquired hospitals, the total number of residents who could be considered as the target group of our survey is 741 individuals. With 139 participants in our survey, we are thus nearly 19% coverage of neurology residents in Germany. This data, including gender distribution (57% female) as well as the obtained median age of 32 years of the participants in this study approximately correspond to data from other published continuing education studies (Biesalski et al. 2018).
